# Eight Years of Collaboration on Biosafety and Biosecurity Issues Between Kazakhstan and Germany as Part of the German Biosecurity Programme and the G7 Global Partnership Against the Spread of Weapons and Materials of Mass Destruction

**DOI:** 10.3389/fpubh.2021.649393

**Published:** 2021-08-09

**Authors:** Lukas Peintner, Edith Wagner, Anna Shin, Nur Tukhanova, Nurkeldi Turebekov, Karlygash Abdiyeva, Olga Spaiser, Yelena Serebrennikova, Erik Tintrup, Andrey Dmitrovskiy, Aliya Zhalmagambetova, Stefan Frey, Sandra Simone Essbauer

**Affiliations:** ^1^Department of Virology and Intracellular Agents, German Centre for Infection Research, Munich Partner Site, Bundeswehr Institute of Microbiology, Munich, Germany; ^2^Section of Experimental Virology, Institute of Medical Microbiology, Jena University Hospital, Jena, Germany; ^3^Central Reference Laboratory, M. Aikimbaev National Scientific Center for Especially Dangerous Infections, Almaty, Kazakhstan; ^4^Center for International Health, Ludwig-Maximilians-University, Munich, Germany; ^5^Deutsche Gesellschaft für Internationale Zusammenarbeit GmbH, Berlin, Germany; ^6^Deutsche Gesellschaft für Internationale Zusammenarbeit GmbH, Almaty, Kazakhstan; ^7^Division OR12 “Chemical and Biological Weapons, Disarmament, G7 Global Partnership”, German Federal Foreign Office, Berlin, Germany; ^8^Bundeswehr Research Institute for Protective Technologies and CBRN Protection, Munster, Germany

**Keywords:** Germany, Kazakhstan, biosafety & biosecurity, surveillance, orthohantavirus, CCHFV, rickettiosis, TBEV

## Abstract

In 2013, the German Federal Foreign Office launched the German Biosecurity Programme with the aim to minimise risks associated with biological substances and pathogens. In this context, the German-Kazakh Network for Biosafety and Biosecurity was established in 2013 and constitutes a successful collaboration between Kazakh and German biomedical organisations, under the co-management of the Bundeswehr Institute of Microbiology (IMB), and the Deutsche Gesellschaft für Internationale Zusammenarbeit (GIZ) GmbH. Ever since then, a network of scientists, stake holders and policymakers has been established, aiming to work on highly pathogenic, potential biological warfare agents with the focus on biosafety and biosecurity, surveillance, detection and diagnostics, networking and awareness raising of these agents in Kazakhstan. Over the past 8 years, the project members trained four PhD candidates, organised over 30 workshops and trainings with more than 250 participants and conducted more than 5,000 PCR assays and 5,000 serological analyses for surveillance. A great success was the description of new endemic areas for *Orthohantaviruses*, the mixture of two *Crimean-Congo haemorrhagic fever virus* genetic clusters, new foci and genetic information on tick-borne encephalitis virus and *rickettsiae* in Kazakh oblasts. The latter even led to the description of two new genogroups. Furthermore, joint contributions to international conferences were made. In this report, we summarise the evolution of the German-Kazakh Network for Biosafety and Biosecurity and critically reflect on the strengths and possible weaknesses. We were able to establish a viable network of biosafety and biosecurity shareholders and to accomplish the aims of the German Biosecurity Programme to lower biosecurity risks by increased awareness, improved detection and diagnostic methods and surveillance. Further, we reflect on forthcoming aspects to lead this interstate endeavour into a sustainable future.

## Introduction

The Republic of Kazakhstan is a landlocked country in Central Asia dominated by continental climate. Totalling up to 2.7 million km^2^, the size of the country approximately is seven times the size of Germany and hosts a relatively small population of 18.6 million inhabitants. Historically, Kazakhstan served as the cradle of many consequential pathogens. Its vast steppes and the hot and dry climate fuelled the evolution of plague (*Yersinia pestis*). Furthermore, diseases like Anthrax, Tularaemia and Brucellosis, to name but a few, are endemic to the region (1–3). There exists a vast amount of under-investigated potential natural foci for the vectors or reservoirs of some of these diseases. Animal burial sites, for instance, may cause spontaneous outbreaks of anthrax after heavy rains or landslides or can be unearthed by digging activities.

Because of this high endemic burden, local authorities in different oblasts (=territories) of Kazakhstan started to establish local so-called Anti-Plague Stations (APS) between 1914 and 1949. At the beginning, the main responsibility of these APS was the epidemiological and epizootic surveillance of plague and of the infection rates with *Yersinia pestis* in their natural hosts, wild living gerbils (*Gerbillinae* spp.), and the vectors, which are fleas (genus *Xenopsylla*) (4). Quickly, the APSs evolved to regional centres for the epidemiological surveillance of other especially dangerous endemic pathogens such as the *tick-borne encephalitis virus* (TBEV), the *Crimean-Congo haemorrhagic fever virus* (CCHFV)*, rickettsiae* and *Orthohantaviruses* (5).

During the Soviet time, Kazakhstan played a role in the research on biological warfare agents and countermeasures. However, after the dissolution of the Soviet Union, Kazakhstan proactively opened all facilities to the public. In 1995, it was confirmed by international observers that all military facilities were shut down and that contaminations were removed.

Furthermore, in 2004 Kazakhstan signed an agreement with the USA to reduce the biological weapons proliferation risk. It is an addendum to the 1995 Nunn-Lugar Cooperative Threat Reduction to prevent the proliferation of biological weapons technology, pathogens and expertise (6). Therefore, today the main biological risk comes from Kazakhstan's rich and diverse natural foci of extremely dangerous pathogens (EDP).

Nevertheless, there are blank spots regarding the spread and epidemiology of EDPs in some areas of Kazakhstan as only limited data is available. Many reports of infections with EDPs in Kazakhstan are based on the clinical presentation of infected patients rather than on contemporary molecular biological and serological diagnostic as part of all-over surveillance studies. In conclusion, frequent fevers of unknown origin in rural and urban residents indicate that more research on the spread of endemic pathogens needs to be conducted. Many major infections display a relatively unspecific clinical picture that often leads to the problem of misclassification of diseases. It is suspected that many cases of infectious diseases caused by EDPs, such as *Orthohantaviruses*, TBEV, and CCHFV, go unnoticed in endemic and so-far non-endemic areas in Kazakhstan.

The Republic of Kazakhstan recognised this issue and actively approached international governmental and non-governmental institutions to modernise its diagnostic capabilities. As part of the U.S. Defense Threat Reduction Agency (DTRA) biological threat reduction programme, the USA funded the improvement of infrastructure, including the construction of the Central Reference Laboratory (CRL) - a BSL3 laboratory in the city of Almaty (6).

There are numerous efforts to support the establishment of a modern biosafety and biosecurity landscape in Kazakhstan together with international partners. Therefore, institutions in Kazakhstan proved as ideal focus points for the new German Biosecurity Programme that was launched by the German Federal Foreign Office in 2013.

## Initiation of the German-Kazakh Network for Biosafety and Biosecurity

In early 2013, the German Foreign Office initiated the German Biosecurity Programme with the aim to foster biosafety and biosecurity for a safer world. This programme was launched within the framework of the German engagement in the G7 Global Partnership Against the Spread of Weapons and Materials of Mass Destruction (WMD) which expanded its activities to questions regarding biological security in 2012 (7). The German Biosecurity Programme is part of the Federal Government's preventive security policy and has the goal to minimise the risks associated with biological substances and pathogens and, therefore, cooperates with selected partner countries worldwide (8). It aims to promote a responsible approach to research and research findings on dual-use pathogens, to strengthen local public health and to prevent a potential menace to Germany. To reach these goals, the project activities are based on six different columns: increasing awareness, networking, capacity development, detection, and diagnostics, surveillance as well as biosafety and biosecurity. The German Initiative of the Federal Foreign Office aims at raising awareness of and minimising the risks associated with highly pathogenic agents, including their potential abuse for the purposes of terrorism. It reached out to German federal laboratories working on different aspects of EDPs and invited them to submit project proposals.

The call reflects part of the mission of the Bundeswehr Institute of Microbiology (IMB), a military research facility of the German Armed Forces for medical biological defence based in Munich. Its task is to develop methods and measures to protect soldiers from diseases caused by biological warfare agents and other dangerous pathogens (9). The focus is on diagnostics, research, teaching, and biosecurity. Due to the institute's interest in especially dangerous zoonotic diseases with natural foci for uncommon outbreaks and their historical significance to Central Asia, Kazakhstan was identified as a partner country to jointly participate in the German Biosecurity Programme.

The landlocked country in Central Asia indeed has several active foci of endemic EDPs, as mentioned above. Furthermore, it has 13.364 km of borders with its neighbouring countries Russia, China, Kyrgyzstan, Uzbekistan, and Turkmenistan and many migratory animals, such as birds or bats, and associated ticks that seasonally can carry new species of pathogens across the country. The additional heavy transport of livestock over far distances further poses the risk of pathogen dispersion e.g., also along the new Chinese Belt and Road Initiative. This diverse and changing EDP landscape is monitored by public health institutions that often reside in aged buildings with equipment still remaining from the Soviet era. The quantity of biosurveillance has decreased in comparison to Soviet times, due to the lack of financial support and the slow establishment of state-of-the art diagnostic tools. The retirement of experienced researchers and the brain drain of younger scientists to more prosperous countries further weakened the input to the Kazakh public health monitoring (10).

In summary, Kazakhstan has all the prerequisites to meet the goals of the German Biosecurity Programme by the Federal Foreign Office. Establishing a network between Kazakh and German biosafety and biosecurity stakeholders will contribute toward raising awareness of and minimising the risks associated with highly pathogenic pathogens, including their potential abuse for the purposes of terrorism. In doing so, it contributes to the G7 Global Partnership Against the Spread of Weapons and Materials of Mass Destruction (WMD) by means of a preventive security-policy.

## The German-Kazakh Network for Biosafety and Biosecurity

The German Biosecurity Programme and the German-Kazakh Network for Biosafety and Biosecurity (GerKazNet) are now in its third funding phase (2013–2016, 2017–2019, 2020–2022). Starting in 2013, the “Asfendiyarov Kazakh National Medical University” (KazNMU) and the “Scientific Practical Center for Sanitary Epidemiological Expertise and Monitoring” (SPC SEEM, 2013) together with the IMB and GIZ were the founding partners of the GerKazNet and the collaboration lasted from 2013–2018. Over the years, the network expanded and welcomed other key players to the Kazakh biosafety and biosecurity framework, such as the “Masgut Aikimbayev's National Scientific Center for Especially Dangerous Infections” (NSCEDI, 2014). The most recent institution, which joined the network in 2019, is the Otar-based “Research Institute for Biosafety Problems” (RIBSP, 2019). It is planned that the “National Center for Biotechnology Branch Almaty” (NCB) will join the network in 2021 as listed in Table 1.

**Table 1:** List of all participants at the German-Kazakh network for biosafety and biosecurity (GerKazNet).

**Name**	**Abb**.	**Year joined GerKazNet**	**Location**	**Belongs to**
Institut für Mikrobiologie der Bundeswehr	IMB	2013	Munich, DEU	MoD
Deutsche Gesellschaft für Internationale Zusammenarbeit GmbH	GIZ	2013	Berlin, DEU, Almaty/Nur-Sultan, KAZ	–
Kazakh National Medical University	KazNMU	2013–2018	Almaty, KAZ	MoH
Scientific Practical Center for Sanitary Epidemiological Expertise and Monitoring	SPC SEEM	2013–2018	Almaty, KAZ	MoH
M.Aikimbayev National Scientific Center for Especially Dangerous Infections^*^	NSCEDI	2014, 2017^**^	Almaty, KAZ	MoH
Scientific Research Institute of Biological Safety Problems	RIBSP	2019	Otar, KAZ	MoES
International Science and Technology Center	ISTC	2020	Nur-Sultan, KAZ	–
National Center for Biotechnology, Branch Almaty	NCB Branch Almaty	2021	Almaty/Nur-Sultan, KAZ	MoES

* *Formerly known as M. Aikimbayev Kazakh Scientific Center of Quarantine and Zoonotic Diseases (KSCQZD).*

** *Active collaborative work started in 2017 in the second project phase.*

*MoD, Ministry of Defense; MoH, Ministry of Health; MoES, Ministry of Education and Science*.

An efficient coordination of such a project demands a professional, well-connected agency that enables a transparent handling of personnel and finances. An important partner in this is the “Deutsche Gesellschaft für Internationale Zusammenarbeit GmbH” (German Corporation for International Cooperation GmbH, GIZ), a federal enterprise and globally active service provider in the field of international cooperation for sustainable development and international education work (11). The GIZ offers consulting and capacity building services in around 120 countries pursuing the goal of permanently improving the living conditions of people worldwide. With its offices in Almaty and Berlin, the GIZ has helped to establish the GerKazNet through legal, financial and consulting services. Furthermore, it has also allowed for a highly professional interaction with policymakers.

The GerKazNet has established a wide network of partners in Kazakhstan. During the project, several reorganisations in ministries and committees took place. Thus, the network regularly exchanged information with the former (2013–2019) Chief Sanitary Doctor of Kazakhstan and the Deputy Head of the former Committee of Quality Control and Goods and Services Security (Safety) at the Ministry of Health which was reorganised in 2020. The board, now called the “Committee of Sanitary and Epidemiological Control of the Ministry of Healthcare” is the national institution to supervise all areas of public health protection including sanitary and epidemiological welfare of the population, as well as the field of food safety, controlling the implementation of regulatory measures. Furthermore, the GerKazNet is in close contact with the Kazakh head of the Department for Coordination of Activities of Scientific Organisations, located at the Ministry of Education and Science.

In 2020, the GerKazNet became a partner of the International Science and Technology Center (ISTC), which is an intergovernmental organisation connecting scientists from several Central Asian countries with their peers and research organisations in the EU, Japan, Republic of Korea, Norway, and the United States. The ISTC facilitates international science projects and assists the global scientific and business community to source and engage with the Commonwealth of Independent States (CIS) and Georgian institutes that develop or possess an excellence of scientific know-how (12).

The role of the KazNMU was to offer the academic surrounding for education in medicine and pharmacy. With regard to that, the molecular biological laboratories were a budding partner in the project during the first two phases of the GerKazNet. The KazNMU is the national medical university, located in Almaty and operated by the Ministry of Health (13). Up to 9,000 students, including 1,000 students from 15 different countries, are enrolled to train for the medical and pharmaceutical profession.

The Ministry of Health also operates the SPC SEEM whose main mission is the surveillance and control of infectious diseases, the detection of epidemics and the recommendation as well as implementation of appropriate countermeasures, as well as providing information to the Kazakh government. For this, the parasitological department collecting vectors throughout Kazakhstan was an important project partner during the first two phases of the GerKazNet.

During the second phase, a close and productive collaboration with the NSCEDI (formerly M. Aikimbayev Kazakh Scientific Center of Quarantine and Zoonotic Diseases, KSCQZD) has been established and is still ongoing. The NSCEDI originated from the “Central Asia Research Anti-Plague Institute” in Almaty, which was founded in 1949 and whose main mission was the epidemiological surveillance of highly pathogenic diseases (anthrax, plague, tularaemia, brucellosis, cholera, listeria, CCHF, and HRFS) and other zoonotic diseases (yersinioses, listerioses, leptospirosis, pasteurellosis, etc.,). The institute is now under the control of the Ministry of Health and is considered a scientific and methodological center for eight anti-plague stations and 15 district departments.

A more recent partner, who joined the network in 2020, is the RIBSP, based in Otar. It conducts research on highly pathogenic viruses and bacteria as well as exotic diseases of farm animals and plants and is subordinate to the Ministry of Science and Education. They establish and mass-produce diagnostic kits and vaccinations against EDPs and other zoonotic agents and animal diseases to support the Kazakh livestock industry. The RIBSP also plays an important role in biosafety, biosecurity, diagnostics and detection of EDPs mainly in animal hosts and vectors. In 2020, it featured prominently in the development of the national COVID-19 vaccine and first clinical tests were announced in August 2020.

To support the development of a biotechnological industry in Kazakhstan, the National Center for Biotechnology (NCB) was established. The NCB carries out government funded scientific and technical programmes in the areas of biotechnology, biosafety, and ecology and translates them into economic endeavours. It is a decentralised institution that operates facilities all over the country with the Branch Almaty focusing on the molecular biological characterisation of newly emerging pathogens.

Part of their research is conducted in the Central Reference Laboratory (CRL) in Almaty. This laboratory was built by the Americans as part of the DTRA activities and contains a state-of-the-art BSL-3 facility. It was ceremonially handed over to the Kazakh government in September 2018 and is jointly operated by three ministries, namely the Ministry of Health *via* the NSCEDI, the Ministry of Education and Science *via* its NCB Branch Almaty and the Ministry of Agriculture with the latter not being a part of the GerKazNet. USA, Great Britain, Germany and Poland actively participated in the training of staff and personnel.

## The Footprint of the Gerkaznet

The purpose of the Global Partnership Against the Spread of Weapons and Materials of Mass Destruction (WMD) is defined by five deliverables that aim at reducing biological warfare threats: (i) control materials that represent biological proliferation risks, (ii) detect and disrupt misuse of biological agents, (iii) improve identification of biological attacks, (iv) reinforce and strengthen Biological and Toxins Weapons Convention (BTWC) practices and instruments, and (v) promote biosafety and biosecurity (7). To reach these goals, the German Biosecurity Programme, coordinated by the German Foreign Office, defined six modes of action: Increasing awareness, biosafety and biosecurity, detection and diagnostics, networking, surveillance, and capacity development. An accompanying toolbox approach containing 25 items was established to help the projects to successfully start their endeavour. The GerKazNet translated these modes of action into several study topics that were put into practice together with the partner institutes.

### Increasing Awareness

To raise sensibility and awareness of issues regarding biosafety and biosecurity, each joint scientific meeting was started by a keynote lecture given by the programme manager or invited speakers on respective topics. On four occasions, scientific lectures on EDPs and on pathogens relevant for medical biodefense and their associated human diseases helped the project partners to identify contact points regarding biosafety and biosecurity. Intense discussions of these issues clarified open questions and set the ground for future projects. Partner institutes and their branches were also informed about the dual-use concept that is applicable to many of the pathogens endemic to Kazakhstan and the importance of blocking access to EDPs to unqualified personnel that could proliferate it to groups with bad intensions.

As part of the programme, an exchange on the importance to share scientific insights on EDPs with the international community was arranged with policy makers at the Ministry of Education and Ministry of Health, in order to enable a bioforensic characterisation that would help to localise the source of future potential illegal use of EDPs. Regular meetings with the former Committee of Quality control of Goods and Services of the Ministry of Health (2013–2019) were used to inform the committee members about the progress of the project and about scientific findings like raising awareness on possible new endemic areas of EDPs but also on legislation in work safety including biosafety and biosecurity. Another key ambition was to raise awareness of the health risks related to some non-endemic pathogens that may migrate to Kazakhstan through birds or enhanced trade resulting from the China's Belt and Road Initiative which could lead to diseases among the residents.

### Biosafety and Biosecurity

A major focus of the project was to organise sustainable trainings on issues of biosafety and biosecurity for staff such as medical doctors, scientists, and lab technicians in small groups. In regular workshops, local staff learned about modern techniques and diagnostic tools with the focus on biosafety and biosecurity. In order to promote a new generation of scientists, four PhD candidates were selected to conduct doctoral studies under the scientific supervision of the IMB in cooperation with the Munich Ludwigs-Maximilians-University and the Center for International Health (CIH) PhD programme. This programme aims to train PhD students from low- and middle-income countries to become the new generation of health care developers in their countries. In this three-year programme, the first part of the education takes place in Munich and the second part in the student's country of origin. The courses in Munich cover topics such as epidemiology, research design and general health issues. Back home in their research laboratory, students are asked to perform scientific research on a selected topic (14). The GerKazNet supported the application of four candidates for this PhD graduate school. They were nominated by the partner institutes in Kazakhstan. All of them had profound medical training and intended to specialise in the area of infectious diseases. During their doctoral research, they implemented the above-mentioned scientific studies. Two of the PhD students successfully graduated in 2019, while the other two will defend their theses in reasonable time. After receiving their PhD degree, the junior scientists got permanent contracts in positions of responsibility at their home institutions. In this role, they contribute to the further improvement of the quality of research and diagnostic services at the institutes and pass on their acquired knowledge to a new generation of researchers following the train-the-trainer principle.

In addition to the complex PhD education, the project aims to give regular training to Kazakh diagnostic and research facilities' staff. In workshops at the partner institutes and anti-plague stations all over the country, small groups ranging from 10 to 12 participants were trained on topics such as hygiene, the safe handling of patient samples, molecular biology, cell culture, the trapping of animals, and next generation sequencing (Table 2). The selection of topics resulted from mutual identification of needs or emphasis by the Ministry of Health. In total 240 technicians and scientists were enrolled in different trainings. The workshops were held in English, Russian, and Kazakh and contained a mixture of theoretical, practical, and applied sessions to coach new methods, diagnostics, and quality assurance under the aspect of biosafety and biosecurity. Group sizes were deliberately kept small to ensure supervised hands-on training in step with actual practice. The quality of the workshops was assessed in pre- and post-tests conducted before and after the training. Due to the high appreciation of these workshops, the GerKazNet intends to continue and expand its training programme, also as online webinars. To this purpose, the GerKazNet hired a dedicated trainer in 2020 who will regularly offer workshops and training units, in order to promote a lasting research culture in Kazakhstan.

**Table 2:** List of all Workshops held at the partner institutes of the GerKazNet.

**#**	**Year**	**Topic of the Workshop**	**Location**
1	2014	Hygiene as a basis of biosafety and biosecurity: disinfection, decontamination, and sterilisation	Almaty
2	2014	Pipetting in diagnostic, research, and regarding biosafety	Almaty
3	2015	International Workshop on molecular biology including next-generation sequencing	Almaty
4	2015	International Workshop on molecular biology	Almaty
5	2015	Modern aspects of parasitological and entomological works in the framework of epidemiological surveillance of tick-borne infections	Almaty
6	2016	International Workshop on molecular biology “Working on RNA”	Almaty
7	2017	Workshop on the safe handling of patient's samples and PCR diagnostics in the field of highly dangerous pathogens	Almaty
8	2018	Workshop on Biosafety and Biosecurity in field studies and highly pathogenic rodent-borne infections	Almaty
9	2018	Workshop on cell culture methods I	Almaty
10	2018	Workshop on cell culture methods II	Almaty
11	2018	Workshop on PCR diagnostics in the field of highly dangerous pathogens	Almaty
12	2019	Workshop on Biosafety and Biosecurity in field studies and highly pathogenic rodent-borne infections	Uralsk
13	2020	Workshops on personal protection in the biological laboratory I	Online
14	2020	Workshops on personal protection in the biological laboratory II	Online
15	2020	Self-learning modules on COVID19 diagnostics	Online

*APS, Anti Plague station*.

### Surveillance, Detection, and Diagnostics

As part of this mode of action, the GerKazNet encourages local scientists to introduce modern diagnostic and detection methods, to perform studies as well as surveillance approaches in their laboratories. In concert with scientists from Germany, they established a bundle of new molecular biologic methods on the prevailing EDPs in Kazakhstan and documented the methods in peer reviewed standard operating procedures (SOPs), that are now routinely used for diagnostic and research in the partner institutions (Table 3). Beyond the scientific output (see chapter below), surveillance data were shared with all partners including stake holders.

**Table 3:** Established serological and molecular biological diagnostic methods by the GerKazNet at KazNMU, SPC SEEM (both partners from 2012 to 2018), and NSCEDI (active partner since 2017).

**Pathogen**	**Method**	**Institute**	**Reference**
*Orthohantaviruses*	Serological (Immunoblot, IIFT, ELISA); molecularbiological (RT-PCR, real-time PCR)	KazNMU SPC SEEM NSCEDI	(15)
CCHFV	Serological (ELISA); Molecularbiologcial (RT-PCR, real-time PCR)	KazNMU SPC SEEM NSCEDI	(16)
Rickettsia (Spotted fever group)	Serological (ELISA, IIFT); Molecularbiological (real-time PCR, PCR)	KazNMU SPC SEEM NSCEDI	(17)
Rickettsia (Typhus group)	Serological (ELISA, IIFT);	KazNMU SPC SEEM NSCEDI	(17)
TBEV	Serological (ELISA, IIFT); molecularbiological (RT-PCR, real-time PCR)	KazNMU SPC SEEM NSCEDI	(18)
Borrelia	Molecularbiological (real-time PCR)	KazNMU SPC SEEM NSCEDI	(19)

*This methods are now routinely in use to facilitate a nationwide surveillance of extremely dangerous diseases in natural habitats.*

*CCHFV, Crimean-Congo Haemorrhagic Fever Virus; SFG, Spotted fever group, TBEV, Tick borne Encephalitis Virus; KazNMU, Kazakhstan National Medical University; NSCEDI, M.Aikimbayev National Scientific Center for Especially Dangerous Infections, here work is performed in the CRL branch of MoH, SPC SEEM, Scientific Practical Center for Sanitary Epidemiological Expertise and Monitoring*.

To ensure an efficient sharing of methods and protocols, and to offer a low-key scientist interaction, the GerKazNet became part of the “German Online Platform for Biosecurity and Biosafety” (GO4BSB), coordinated by the “Bernhard Nocht Institute for Tropical Medicine” (BNITM) in Hamburg, Germany. This platform is run within the framework of the German Biosecurity Programme, is open to all partners, and serves as a depository for methods, protocols, background information and training material. Incorporating the use of this platform into the workshops opened it up to a broad audience. It is the aim to establish the GO4BSB platform as the central hub of Kazakh zoonosis scientists when it comes to the local sharing of information, methods and ideas (20).

### Networking

International perception of the GerKazNet-activities was successfully increased by jointly attending international and national conferences, in order to present the progress of individual scientific projects, but also the goals of the GerKazNet itself. Members of the project presented the progress of their research in 41 oral talks and 21 poster presentations (Table 4). Due to the continuous attendance of conferences, the German-Kazakh initiative became recognised as a reliable partner in the Central Asian area. This led to an active participation in the organisation of the “Biosafety Association for Central Asia and the Caucasus” (BACAC) conference in 2019.

**Table 4:** List of all conferences jointly visited by scientists of the GerKazNet.

**Year**	**Name of Conference**	**Location**	**Participating partners of the GerKazNet**	**Co-organisation by the GerKazNet**
2013	Medical Biodefense Conference	Munich, DEU	IMB KazNMU	No
2013	German Symposium on Zoonoses Research 2013	Berlin, DEU	IMB	No
2014	German Symposium on Zoonoses Research 2014 and International conference on Emerging Zoonoses	Berlin, DEU	IMB KazNMU	No
2014	Meeting of Parasitologists	Almaty, KAZ	IMB GIZ SPC SEEM	No
2014	The first international scientific-practical conference of the Agency of the Republic of Kazakhstan on the protection of consumer rights, dedicated to the 100 anniversary of the anti-plague service of the Republic of Kazakhstan and the Ural anti-plague station”	Uralsk, KAZ	IMB GIZ NSCEDI APS Uralsk	No
2014	ASM Biodefense and Emerging Diseases	Washington D.C., USA	IMB	No
2014	The Future of Biosafety and Biosecurity in Central Asia and further countries from the region	Bishkek, KGZ	IMB GIZ	Yes
2014	Kongress für Infektionskrankheiten und Tropenmedizin,	Cologne, DEU	IMB GIZ	No
2015	ASM Biodefense and Emerging Diseases	Washington D.C., USA	IMB	No
2015	National Symposium on Zoonoses Research 2015	Berlin, DEU	IMB	No
2015	Biostudy Tour	Munich, Berlin, DEU	IMB GIZ MoES MoH SPC SEEM	Yes
2016	ASM Biodefense and Emerging Diseases	Washington D.C., USA	IMB	No
2016	Roundtable MRI-Global, DTRA, ASM Biodefense	Washington D.C., USA	IMB	No
2016	National Symposium on Zoonoses Research 2016	Berlin, DEU	IMB KazNMU	No
2016	Congress “Diagnosis and prevention of infectious diseases at the present stage”	Novosibirsk, RUS	IMB KazNMU	No
2016	Actual Problems of epidemiology, microbiology, and natural foci of human diseases	Omsk, KAZ	IMB KazMNU	No
2016	Medical Biodefense Conference	Munich, DEU	IMB GIZ MoH NSCEDI SPC SEEM	Yes (session)
2016	Symposium des deutsch-kasachischen Netzwerkes zur Diagnostik von gefährlichen Infektionskrankheiten	Almaty, KAZ	IMB GIZ MoH KazNMU SPC SEEM NSCEDI SES DTRA CDC	Yes
2016	Kazakh Youth Forum, Presidents Roundtable at Presidents Day - 25 years of Kazakhstan's Independence	Almaty, KAZ	IMB KazNMU	No
2017	ASM Biothreats, Research, Response and Policy	Washington D.C., USA	IMB	No
2017	International Conference “Current Issues on Zoonotic Diseases”	Ulan-Bataar, MNG	KazMNU	No
2017	Scientific Practical Conference	Nur-Sultan, KAZ	IMB GIZ KazNMU	Yes
2017	National Symposium on Zoonoses Research 2017	Berlin, DEU	IMB GIZ KazNMU	No
2017	Meeting of the State Parties to the Convention on the Prohibition of the Development, Production and Stockpiling of Bacteriological (Biological) and Toxin Weapons and on their Destruction	Geneva, CHE	IMB	No
2018	ASM Biothreats	Washington D.C., USA	IMB	No
2018	The third Baikal International Scientific Conference on vector-borne natural foci infections dedicated to the 100th anniversary of the Irkutsk State Medical University Foundation	Irkutsk, RUS	IMB KazNMU	No
2018	National Symposium on Zoonoses Research 2018	Berlin, DEU	IMB	No
2018	Regional Workshop for Central Asian States Parties to the Biological Weapons Convention on scientific and practical implementation issues	Almaty, KAZ	IMB CRL/NSCEDI	Yes
2018	International Scientific Symposium “Emerging infections: Increasing preparedness by networking”	Berlin, DEU	IMB	No
2018	Medical Biodefense Conference	Munich, DEU	IMB GIZ MoH MoES NSCEDI KazNMU	Yes
2019	EU CBRN Centres of Excellence - Biosafety Association of Central Asia and the Caucasus Conference BACAC: Bridging the Gaps	Tashkent, UZB	IMB GIZ	Yes
2019	International Conference “Current Issues on Zoonotic Diseases”	Ulaanbaatar, MNG	IMB	No
2019	LMU, Center for International Health: Occupational Safety & Health Symposium 2019	Munich, DEU	IMB KazNMU	No
2019	International Scientific Conference “Dangerous infections: new solutions – a look into the future”	Almaty, KAZ	IMB NSCEDI GIZ	Yes
2019	Travelling conferences on actual distribution, modern detection methods and aspects of biosecurity for highly pathogenic zoonotic agents in Central Asia and Mongolia	Almaty, KAZ/Duschanbe, TJK	IMB NSCEDI 16 APS	Yes
2021	Online Symposium “COVID-19 and other emerging zoonoses”	Online	IMB GIZ NSCEDI RISBP NCB	Yes

*IMB, Bundeswehr Institute of Microbiology; KazNMU, Kazakhstan National Medical University; NSCEDI, M.Aikimbayev National Scientific Center for Especially Dangerous Infections, SPC SEEM, Scientific Practical Center for Sanitary Epidemiological Expertise and Monitoring; GIZ, Gesellschaft für Internationale Zusammenarbeit GmbH; APS, Anti Plague Station; MoH, Ministry of Health; MoES, Ministry of Education and Science; CRL, Central Reference Laboratory Almaty; SES, Sanitary Epidemiology Stations; DTRA, Defense Threat Reduction Agency; CDC, Centers of Disease Control and Prevention*.

The participation of Kazakh scientists at a series of travel conferences on the distribution, modern detection methods and aspects of biosecurity for highly pathogenic zoonotic agents was also highly valued. These events were co-funded by the BMBF and the German Research Platform for Zoonoses and were held four times in four different cities - Dushanbe (Tajikistan), Ulaanbaatar (Mongolia), Munich (Germany), and Almaty (Kazakhstan) - to discuss actual issues of biosafety and biosecurity (21, 22).

## Scientific Output of the Project

### Peer Reviewed Publications

**Figure 1:**
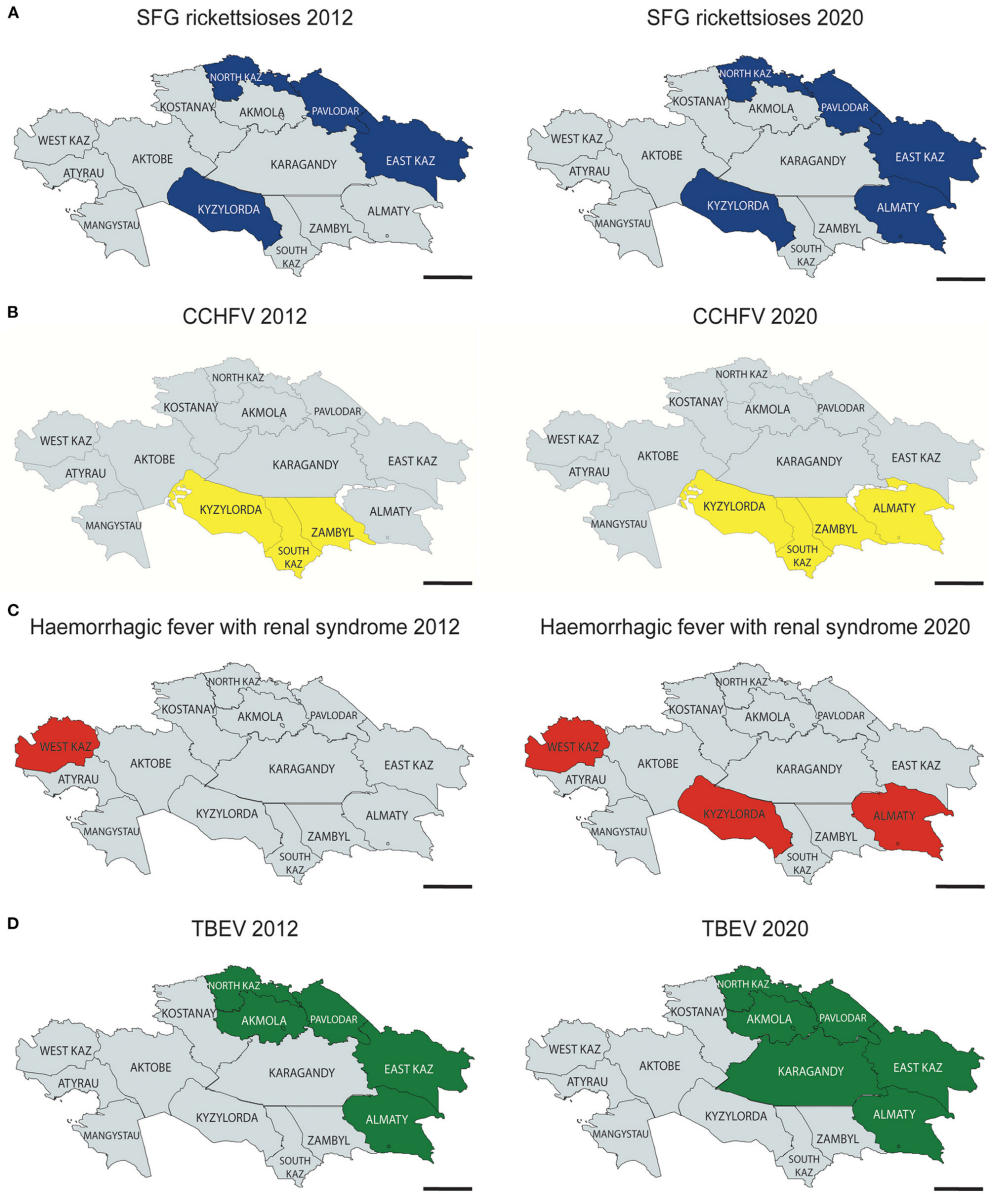
Geographical overview of Kazakhstan and pathogens known to be endemic. Maps summarising the distribution of endemic areas for extremely dangerous pathogens (EDP) in Kazakhstan as known by 2012 (left panel) and 2020 (right panel). Pathogens investigated in the project were Rickettsia (Spotted Fever Group, SFG) **(A)**, *Crimean-Congo Haemorrhagic Fever Virus* (CCHFV) **(B)**, *Orthohantavirus* (Haemorrhagic fever with renal syndrome) **(C)**, and *Tick Borne Encephalitis Virus* (TBEV) **(D)**. Scale = 500 km.

The characterisation of new and emerging diseases caused by highly pathogenic agents or biological warfare agents in Kazakhstan and the publication of these scientific findings in peer-reviewed journals constitutes great publicity for the GerKazNet and also increases the sustainability its achievements. As described above, the GerKazNet employed four postgraduate students to explore so far under-investigated foci for *Tick Borne Encephalitis Virus* (TBEV), *Crimean-Congo haemorrhagic fever virus* (CCHFV), *Orthohantaviruses* (OHV), and *Rickettsia* in Kazakhstan (Figure 1).

In order to get an impression of the distribution of these four selected tick- and rodent-borne highly pathogenic agents in natural foci in Kazakhstan, several surveillance studies were conducted. This was done with the aim to prevent infections and to improve countermeasures against the possible natural spread of zoonotic - but also biowarfare - agents always including the aspects of biosafety and biosecurity.

The tick borne encephalitis virus (TBEV) causes a devastating disease in humans and is transmitted *via* the bite of ticks. TBEV cases are described in Kazakhstan. However, it is still a matter of debate as to how this virus migrated to and spreads in Kazakhstan. To determine the prevalence and circulating subtypes of TBEV in the oblasts of Almaty and Kyzylorda, more than 2.300 ticks were screened for the virus. The sequencing of E-genes of the virus revealed that Kazakh TBEV samples belong to two different clades of the Siberian subtype (Figure 1D). Three endemic tick species are responsible for the transmission of the virus (18). The results of these investigations were recognised by the Ministry of Health in Nur-Sultan. As a consequence, the ministry also commissioned a survey of new suspected foci of TBEV, e.g., in the Akmola oblast. These investigations are still in progress and are in accordance with the goals of the GerKazNet.

A second study on vectors focused on the prevalence of *Rickettsia* species in the above-mentioned ticks in two pilot regions in Kazakhstan. More than 2.300 ticks were screened by RT-PCR for DNA of spotted-fever group *Rickettsia* (17). This screen reported the prevalence of four Rickettsia species: *R. raoultii, R. slovaca, R. yenbekshikazahkensis*, and *R. talgarensis*. In the course of this, two new Rickettsia genotypes *R. yenbekshikazahkensis* and *R. talgarensis* were found that were new to taxonomists. This study also confirmed *Rickettsia*-positive ticks in the endemic region of Kyzylorda and highlights the prevalence of rickettsiae in the non-endemic area of Almaty (Figure 1A). In the last 25 years, over 4,000 persons suffered from rickettsioses in Kazakhstan, but no cases are reported from the region of Almaty (23). Furthermore, this new insight into the spread of *Rickettsia* in ticks highlights how incomplete the knowledge of the spread of *Rickettsia* in Kazakhstan currently is, and that there is a demand for a closely monitoring for *Rickettsia*, not only in Kazakhstan but the entire Central-Asian region.

Small mammals such as rodents, insectivores and bats are the reservoir for *Orthohantaviruses*. There is only limited data available from *Orthohantaviruses* in small mammals from Uralsk (Figure 1C). A still ongoing surveillance study in the GerKazNet is the investigation of wild rodents, such as voles, mice and rats, as vector or reservoir for *Orthohantaviruses* and also *Rickettsia*. The aim of this study is to investigate the *Orthohantavirus* diversity in rodents in three areas: in West Kazakhstan, Almaty Oblast and Almaty city. This study will - for the first time - reveal the occurrence and prevalence of *Orthohantaviruses* in wild living rodents.

Small mammals were also found to harbour rickettsial DNA in ear tissue. The analysis of a broad species panel of small mammals will show which *Rickettsia* occur in the area of western and south-eastern Kazakhstan. These studies also help to further coordinate attempts of timely anti-epidemic measures in Kazakhstan.

In addition to these surveillance studies in vectors and reservoirs, several studies in human patients were conducted. The first of these took place in 2014–15 on patients with fever of unknown origin in 13 hospitals in Almaty and Kyzylorda oblast. Fevers of unknown origin (FUO) are frequently occurring in low and middle income countries and are often a result of incomplete diagnostic setups (19, 24).

CCHFV causes severe forms of haemorrhagic fevers in humans. First documents of CCHFV exist since 1948 in Kazakhstan and it had an average lethality rate of 15%. So far, however, the description was limited to Kyzylorda, Zhambyl and South-Kazakhstan oblast (25). To get a contemporary picture of the situation in Kazakhtsan, a study was carried out which compared FUO patients in the non-endemic area Kyzylorda with patients from the endemic area Almaty. This investigation proved that CCHFV is much more distributed than previously assumed (16). About 13% of sera from 802 patients with FUO contained IgG antibodies against CCHFV that gives evidence about a previous CCHFV infection. 0.87% of the patients had an acute infection. Importantly, this rate was also found in the non-endemic area of Almaty (Figure 1B). Molecular biological analysis of viral RNA in the serum samples was successful and specified as Asia 1/2 subtypes of CCHFV. The explanation for the expansion of endemic regions for CCHFV to the south-western oblasts of Kazakhstan might rest in the high density of the population in this area and the high migratory potential of the *Hyalomma* ticks that serve as a vector for CCHFV. These ticks migrate on birds and animals in and out of neighbouring endemic areas such as Uzbekistan and Tajikistan. Endemic regions in Kazakhstan recorded 119 cases of CCHF in the years 2000–2013. This number ignores all FUO events in Almaty Oblast. Future FUO patients of the Almaty region will now be systematically screened for CCHFV infections (16).

Furthermore, the sera from FUO patients described above were examined for the presence of *Orthohantavirus* (OHV)-specific antibodies. HFRS caused by *Orthohantavirus* infection is frequently registered in the endemic areas of West Kazakhstan, but may also occur in non-endemic areas in Kazakhstan. A similar conclusion could be drawn from this OHV study. In more than 800 FUO patients sera, about a fifth had antibodies against *Orthohantaviruses* (15). Further, serotyping characterised the genotypes *Puumala orthohantavirus* (PUUV), *Hantaan orthohantavirus* (HTNV) and *Dobrava-Belgrade Orthohantavirus* (DOBV) for the Almaty region, and PUUV and DOBV for the Kyzylorda region – both regions that were previously considered non-endemic for *Orthohantaviruses* (26). Currently, only West Kazakhstan Oblast is declared an endemic area for *Orthohantaviruses* and so only there do practitioners have access to diagnostic tools that enable a proper detection of the viral infection (Wagner and Tukhanova et al., in revision). Doctors and patients in other oblasts do not have access to *Orthohantavirus*-laboratory diagnostics and have to rely on differential diagnosis. Therefore, this demands the building of awareness of this disease among doctors and the public (Figure 1C).

Thirdly, the serum samples from the FUO patients from Almaty and Kyzylorda region were examined for the occurrence and prevalence of spotted-fever group and typhus group *Rickettsia* antibodies (27). So far, Rickettsioses are not on the routine check lists for patients with FUO in Kazakhstan. The data reveal that about 30% of all tested sera of patients showed antibodies against spotted fever group and typhus group *rickettsiae*. This is the first time that past and acute rickettsia infections were diagnosed in Kazakh residents and it highlights the need to further investigate the distribution of *Rickettsia* in Kazakhstan.

In the next phase of the project, two studies on acute infections with some of the agents were initiated. To visualise TBEV infections of humans with tick bites, a study in hospitals in Almaty and Kyzylorda will be performed. Sera and spinal fluid of patients developing a meningitis or meningoencephalitis will be screened for TBEV antibodies. This project will help to establish a reliable TBEV diagnostic in Kazakh laboratories by implementing further, more modern serological and molecular biological assays.

A collection of samples for the examination of *Orthohantaviruses* with regard to their role concerning infections in acute patients was performed in eight hospitals in 2018 and 2019. Included in the study were patients presenting with either fever and/or abdominal pain and/or feeling seriously ill and/or with renal insufficiency (19). The results of this study will be obtained in the following phase of the project.

### Textbooks and Monographies

Besides making possible basic science investigations, the German-Kazakh Network also has seen to the realisation of a textbook by Alim Masgutovich Aikimbayev, MD (28). In this textbook titled “The biological safety system in Kazakhstan,” the Almaty based scholar summarises the current knowledge on endemic infectious diseases in Kazakhstan, possible medical, ecological and socio-economic consequences of this diseases and gives an in-depth insight into the Kazakh biosafety and biosecurity landscape. This book was published in two languages, Russian and English, to bring the information to a broad national and international audience.

## The GerKazNet – A Success Story?

Since 2013, in its eight years of existence, the GerKazNet was able to set a prominent mark in the biosafety and biosecurity landscape of Kazakhstan. Constant efforts in raising awareness on topics regarding biosecurity, biosafety, dual-use and EDPs established a lasting expertise at Kazakh public health institutions. It was possible to translate this expertise into a notable scientific output. In at least five publications and several more to come, many white spots of pathogen diversity in Kazakhstan were erased. Surveillance studies were initiated and carried out to investigate tick-borne encephalitis, haemorrhagic fevers and rickettsioses in natural foci. Furthermore, patients with FUO were examined and the causative agents of their illness was identified in many cases. The studies on acute patients were important for the establishment of modern techniques for the diagnostics of the respective disease. Regular reporting of the scientific insights to policy makers kept the issue of bio surveillance on the top of the political agenda. This may have a direct impact on the treatment quality of infected patients and increase the life quality of the local population in the long term. The results from our investigations also highlighted the need for further surveillance studies - supported also by the MoH - to investigate the spread of diseases such as tick-borne encephalitis, haemorrhagic fevers and rickettsioses in natural foci in other areas of Kazakhstan.

The high quality publications were achieved by the excellent cooperation of all the stakeholders of the GerKazNet. In addition to providing state of the art BSL-2 laboratory equipment, a massive effort was put into educating and training staff and scientists on state-of-the-art diagnostic procedures and new means of pathogen detection methods in the context of biosafety and biosecurity. This training campaign was well-received and highly demanded and all participants showed effective gain in knowledge. For all milestones, a strict project monitoring is done by the GIZ in cooperation with the Federal Foreign Office. Internal monitoring is performed by weekly short reports of the PhD students, by presentations at least four times a year at consultant meetings and by annual reports.

Top-class scientific output of the four PhD-graduates, who received excellent training at the CIH-LMU international graduate programme, will also be maintained in the future. Their experience will have a lasting impact on the surveillance, detection, and diagnostic capacities of pathogens and further raise awareness of issues of biosafety and biosecurity in the country. Furthermore, they are now familiar with applying for international scientific funding and the proper handling of the publishing process, which will help the Kazakh scientific community to further thrive and become more visible internationally.

A bilingual approach has been constantly chosen for all of the above mentioned activities. In some cases, when requested, trainings were also conducted in the Kazakh language. The employment of simultaneous translation brought about fruitful discussions across language barriers and so further promoted the shaping of international collaborations.

## Challenges in the Past

Operating such a global network of scientists and stakeholders naturally poses several challenges that need to be addressed in order to establish a smooth progress.

Scientists in Kazakhstan are used to cooperate with laboratories from all over the world. In the last 30 years, many collaboration projects were put in place with either Russia or the United States of America. The US American way of collaboration focussed on the development of infrastructure (such as the construction of the BSL-3 laboratory in Almaty in 2015) and large-scale training (6). In comparison, the German scientific interaction focuses on the personal development of human resources. Financial aid is always coupled with the accomplishment of clearly defined goals, such as publications or obtained degrees, and measurable changes in the work routine at the laboratories according to biosafety and biosecurity principles.

Besides the challenges of establishing cross-border joint scientific projects, the GerKazNet also made a great effort to make the best out of the intercultural challenges. With the employment of key management personnel that understands both mentalities, an efficient communication was established. Designated consultants at each partner institute maintained an efficient and direct communication. Over the years, the network grew considerably and reliable communication was the basis to build trust. This communication was eminently professionalised by the involvement of the “Deutsche Gesellschaft für Internationale Zusammenarbeit (GIZ) GmbH” and its country office in Almaty in the GerKazNet. The GIZ staff operates a well-established network and is familiar with Kazakh routines and traditions. Thus, a local project coordinator ensured clear, effective and coordinated communication that is critical to the successful implementation of the project. As a result, the GerKazNet has built and maintained good rapport with all target groups, including its strategic partners in Kazakhstan such as the scientific institutions, but also the involved Kazakh ministries, the German embassy and the German General Consulate, the project consultants and the project team.

Communication was also the key to lead the organised events to success. All the joint conferences, workshops, field trips, trainings and participations in events of the partner institutions were only possible through regular concertation on ideas and needs. Hence, due to the nature of work at the partner institutions, all events organised by the programme needed to be coordinated much in advance.

It is also worth mentioning that the scientific results published in the programme's framework also started a political debate. Identifying new areas of highly infectious pathogens naturally triggers a reaction of the local authorities (Figure 1). Before Kazakh regional medical persons in charge commissioned any changes in their surveillance policy, they intensely scrutinised the publications of the GerKazNet. This step was very legitimate, since changes in healthcare policy are cost intensive. However, since all the published results reached high levels in quality and since all the necessary controls and a gapless documentation of all the cases existed, policymakers trusted the results of the project and initiated appropriate steps. This acceptance even led to the request by the Ministry of Health to, e.g., further, investigate the distribution of TBEV in the area of Pavlodar.

Despite all the efforts to maintain a transparent bilingual communication, the language barrier remained a constant challenge. In general, the English language is taught at schools. Still, not every young Kazakh citizen speaks or understands English, let alone persons who received their education in the former USSR. It was utterly important for the project that the recruited PhD students for the International PhD programme were fluent in English as this was a prerequisite to attend the classes. In addition, scientists in advanced positions often were not fluent in English. This was especially challenging when it came to summarise research results in order to publish them in international peer-reviewed journals. For joint meetings, the network maintained the service of real time translation between German and Russian by an interpreter to avoid potential pitfalls and misunderstandings. A thorough knowledge management in the form of clear communication and documentation of all decisions reached jointly was also important, since there was a frequent fluctuation of personnel in the German and the Kazakh delegation. Clearly defined project goals and written roadmaps helped new members of the team to quickly grasp the concept and proceed with the project.

Nevertheless, the efficient management of scientific analysis was very extensive since there are strict Kazakh governmental regulations on the transport, export and import of biological specimens. According to Kazakh law, biological samples generated in Kazakhstan are not allowed to leave the country. This drawback actually turned in a gain for the project: since it promoted the exchange of methods and diagnostic expertise between Kazakhstan and Germany, the motivation of local scientists was increased, since they were able to completely conduct the pipeline of research in their home institution from collecting the specimens, to isolation and analysis of the results. However, it was not possible to use the equipment of the IMB as a backup reference laboratory in cases of non-specific diagnostic results obtained in a Kazakh laboratory. Transportation of supply material from Germany to Kazakhstan was solved by the full support of the ISTC gained by the new partnership.

## Outlook

Over the last 8 years, the GerKazNet, run by the German Biosecurity Programme as part of the G7 Global Partnership group, established new means of scientific and personal interaction in both Kazakhstan and Germany, to promote issues of biosafety and biosecurity. The close cooperation has led to a synergy effect. While learning to trust each other's capabilities and recognising each other as equivalent partners, it was possible to gain new scientific insights on the spread of highly infectious diseases. By implementing the capacity development approach by the German Biosecurity Programme, international safety standards on biosafety and biosecurity were adopted in all the partner institutes and risk identification and risk management were put to a contemporary level. Furthermore, by fully exploiting its toolbox, the German Biosecurity Programme contributed to the reinforcement and strengthening of biological non-proliferation principles and practices, and to the reduction of the risk of proliferation through the advancement and promotion of safe and responsible conduct in the biological sciences.

The unanticipated outbreak of SARS-CoV-2 starting in early 2020 brought topics such as biosafety and biosecurity into the minds of the general public. In his speech to the 75th General Debate of the UN General Assembly in September 2020, the president of Kazakhstan, Kassym-Jomart Tokayev, proposed the establishment of a biological weapons control system in light of the global COVID-19 pandemic. The proposition entailed the establishment of a special multilateral body – the International Agency for Biological Safety – based on the 1972 Biological Weapons Convention and accountable to the UN Security Council. Furthermore, he suggested to closely examine the idea of a network of Regional Centers for Disease Control and Biosafety under the UN auspices and expressed Kazakhstan's readiness to host such a regional center (29). This initiative reflects Kazakhstan's commitment to the field of biosecurity and may give new momentum to the stalled negotiations on the implementation of the BWC goals. Furthermore, it would sustain and accelerate the knowledge of biosafety, biosecurity and dual-use that was initiated by the GerKazNet among others.

Eight years of GerKazNet brought together scientists and other stakeholders from all over the country. Scientists from different institutions became partners and ultimately trusted friends and started projects outside of the GerKazNet. This establishment of a stable network nourishes the hope that a close interaction between the scientists and institutes will have a long lasting impact on the Kazakh scientific landscape stretching far beyond the third project phase in 2022.

## Author Contributions

LP, EW, and SE conceived the layout of the project. LP and EW wrote the manuscript. EW and LP created the figures and tables. AS, NTuk, NTur, KA, OS, YS, ET, AD, AZ, and SF contributed additional information and reviewed the manuscript. SE and LP supervised the project and were in charge of the revision process. All authors contributed to the article and approved the submitted version.

## Author Disclaimer

Opinions, interpretations, conclusions and recommendations are those of the authors and are not necessarily endorsed by Bundeswehr Joint Medical Service or any other governmental institutions.

## Conflict of Interest

OS was employed by company Deutsche Gesellschaft für Internationale Zusammenarbeit GmbH. The remaining authors declare that the research was conducted in the absence of any commercial or financial relationships that could be construed as a potential conflict of interest.

## Publisher's Note

All claims expressed in this article are solely those of the authors and do not necessarily represent those of their affiliated organizations, or those of the publisher, the editors and the reviewers. Any product that may be evaluated in this article, or claim that may be made by its manufacturer, is not guaranteed or endorsed by the publisher.

## Abbreviations

APS: Anti-Plague Station
BACAC: Biosafety association for central Asia and the Caucasus
BMBF: German Ministry of Education and Research
BSL: Biosafety level (1–3)
B(T)WC: Biological (and Toxins) Weapons Convention
CCHFV: *Crimean-Congo Haemorrhagic Fever Virus*
DOBV: *Dobrava-Belgrade orthohantavirus*
EDP: Extremely dangerous pathogens
GerKazNet: German-Kazakh Network for Biosafety and Biosecurity
FUO: Fever of unknown origin
HTNV: *Hantaan orthohantavirus*
HFRS: Hemorrhagic fever with renal syndrome
IgG: Immunoglobuline G
IgM: Immunoglobuline M
OHV: *Orthohantavirus*
PUUV: *Puumala orthohantavirus*
RT-PCR: Reverse transcription Polymerase chain reaction
SEOV: *Seoul orthohantavirus*
SES: Sanitary Epidemiology Station
TBEV: *Tick borne Encephalitis Virus*
WKR: West Kazakhstan Region
WMD: Weapons and Materials of Mass Destruction.
